# Percutaneous tracheostomy in COVID patients. Experience in our hospital center after one year of pandemic and review of the literature

**DOI:** 10.4317/medoral.24826

**Published:** 2021-08-19

**Authors:** Jorge Vallejo-Díez, Beatriz Peral-Cagigal, Claudia García-Sierra, Marina Morante-Silva, Luis Antonio Sánchez-Cuellar, Luis Miguel Redondo-Gonzalez

**Affiliations:** 1 Oral and Maxillofacial Surgeon in Hospital Universitario Rio Hortega, Valladolid

## Abstract

**Background:**

The pandemic caused by SARS-COV-2 has caused an increase in the need of tracheostomies in patients affected with respiratory distress syndrome. In this article we report our experience during a year of pandemic, we develop our surgical technique to perform percutaneous tracheostomy with the patient in apnea and we compare our results with those of other centers through a bibliographic review.

**Material and Methods:**

A one-year retrospective clinical study was carried out on tracheotomies performed on patients admitted to the intensive care unit with severe SARS-CoV-2, with difficulty for ventilation or weaning. The technique performed was percutaneous, with fibroscopic control through the endotracheal tube, keeping the patient under apnea during the opening of the airway, reducing by this method the risk of exposure to the virus.

**Results:**

From 35 percutaneous tracheotomies performed, 31% of the patients died from respiratory complications due to SARS-COV-2, but none due to the surgical procedure. The most frequent complication (8.5% of patients) was bleeding around the tracheostoma, resolved with local measures. No healthcare provider involved in the performance of the technique had symptoms or was diagnosed with COVID-19.

**Conclusions:**

Our technique of performing percutaneous tracheostomy maintaining apnea during the procedure, under fibroscopic control, has proven to be safe for all those involved in the procedure, and for the patient.

** Key words:**Percutaneous tracheostomy, coronavirus, COVID-19, SARS-CoV-2.

## Introduction

In early December 2019, an outbreak of patients with pneumonia of unknown origin began to emerge in the city of Wuhan, China. The sequence of its genome showed that this severe acute respiratory syndrome (SARS) was caused by a coronavirus, known as SARS-COV-2 (1).

The first infection registered in Spain dates from January 31, 2020, in a German patient admitted to Hospital of La Gomera (Canary Islands). At the end of March, 275,434 cases were globally confirmed, with 11,299 deaths (2).

On April 12, 2020, as it was reflected by the Spanish Ministry of Health, cases in the country reached 161,852, the highest number of infected in Europe.

In the city of Valladolid, the first case dates from February 27, in a patient of Iranian origin, but it was not until March 12 when a significant increase in cases began to be registered, reaching its maximum on April 12 with 234 patients diagnosed that same day.

According to the data, approximately 10% of these patients required admission at the intensive care unit (ICU) or reanimation unit (REA), and 5% invasive mechanical ventilation (IMV). In order to facilitate the weaning process from IMV, or with the aim of reducing or withdrawing sedation, the need for a tracheostomy is common. In addition, tracheostomy reduces airway resistance, decreasing work of breathing and optimizing control of tracheal secretions (3).

In general, in patients who remain intubated and those who are expected to require mechanical ventilation for a long period of time, a tracheostomy is indicated to avoid future complications such as tracheal or subglottic stenosis (4).

In this article we have carried out an observational, descriptive and retrospective study about the performance of percutaneous tracheotomies in ICU / REA patients infected by SARS-COV-19 in our center, during the first year of the pandemic.

## Material and Methods

This is a descriptive and retrospective study of patients suffering from SARS-CoV-2, intubated and with IMV, on whom we have performed percutaneous tracheostomy, mainly from our province (Valladolid), and from other cities in Castilla y León, as the Rio Hortega Hospital in Valladolid is a reference center for ECMO therapy (extracorporeal membrane oxygenation).

We registered 35 tracheostomies performed in our hospital in the period of time between March 14, 2020 and March 14, 2021. The variables that were analyzed were: age, sex, days of IMV until tracheostomy, type of tracheostomy, complications of the intervention, decannulation and time until it, treatment with ECMO therapy, and days of admission until healing or death. All data were included in Microsoft Excel and a statistical study was carried out using the SPSS Version 15.0 program (SPSS Inc, 1989-2006).

In that period in which we realized the study, a total of 2119 admissions with a SARS-CoV-2 diagnosis were recorded, of which 184 were admitted to intensive care units (ICU or OR) (Table 1)

According to the protocol of our center, the patients initially received treatment with non-invasive mechanical ventilation, progressing to orotracheal intubation in those cases in which an adequate oxygen saturation could not be maintained, and performing tracheostomy in those it was pretended to perform a decrease in sedation or weaning from sedation or IMV, or those subjected to a prolonged intubation, without forecast of extubation in a short period of time.

The patients were followed up from the time the tracheostomy was performed until the patient was discharged from the hospital, death or the end of the follow-up of our study (April 14, 2021).

- Surgical technique

The location to perform the tracheotomies, in our case, was directly in the ICU beds or in the operating room in the case of patients admitted to Anesthesia Unit, in the same bed where the patient was admitted. In most cases, during the SARS outbreak, tracheotomies were performed in ICU beds, preferably in negative pressure rooms (5). This avoids a displacement of the patient, and a repeated connection and disconnection of the ventilation equipment.

**Table 1:**
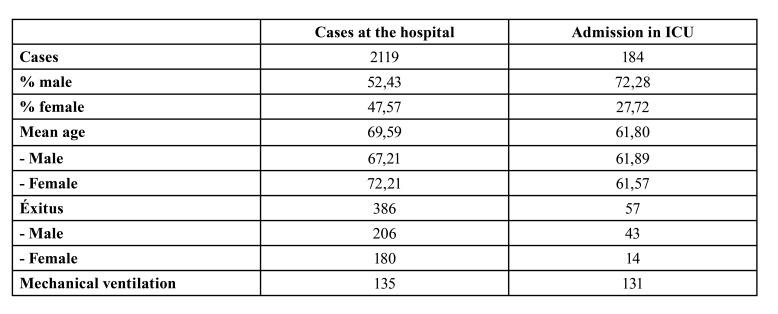
Numbers of cases observed at our hospital and admitted to ICU.

However, depending on each center and the disposition of each hospital, it is strongly suggested to increase individual protection measures and reduce the number of staff during the procedure to avoid the number of infections as much as possible (6).

Following recommendations of the Spanish Society of Oral and Maxillofacial Surgery, as personal protective equipment (PPE), we use: FFP3 (N95) mask under a surgical mask, surgical cap, full protection goggles, waterproof diver hood, splash screen, Wwaterproof gown, waterproof leggings and two pairs of gloves.

The team that performs the procedure consists of two maxillofacial surgeons, who perform both as surgeons (perform the tracheostomy) and as instrumentalists, an anesthetist or intensivist who is in charge of removing the orotracheal tube at the time of the tracheostomy, under fibroscopic supervision, and a nurse and an assistant who assist the intensivist and prepare / administer the medication indicated by the doctor in case more sedation is required.

The type of tracheostomy and the cannula used were: Portex® ULTRAPerc® number 8 percutaneous dilatation tracheostomy kit, adjusTable in length according to the anatomical characteristics of the patient (cervical length and thickness).

In our case, it was decided to perform a percutaneous tracheostomy because it has several advantages over the conventional open one:

1- Performed in the critical box itself, without displacing the patient to the operating room.

2- The average time to perform the technique does not exceed two minutes.

3- The percutaneous technique does not limit the use of cannulas of any diameter gauge.

4- It does not require additional surgical instruments or coagulation systems that could generate aerosols.

5- This technique ensures the trachea-cannula seal, minimizing peritracheostoma leakage.

6- It does not require postoperative cures or requires suturing any the surgical wound.

7- Simple replacement of the tracheostomy tube from the first week.

- Surgical sequence of the percutaneous tracheostomy used in our center

**Figure 1:**
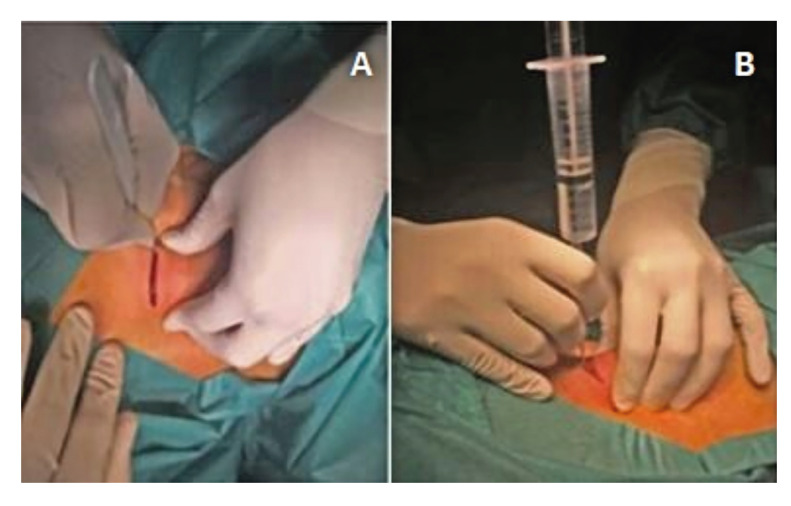
A. Cervical incision; B. Puncture between 2nd-3rd tracheal ring.

1- Placement of the patient in a supine position, with a roller under the shoulders and head in hyperextension. The incision line is marked. The area is disinfected and sterile drapes are placed. Holding the trachea with the hand that is not holding the scalpel. Horizontal incision and blunt dissection to the tracheal surface with curved Mosquito-type forceps (Fig. 1).

2- The intensivist / anesthetist will stop ventilation of the patient (leaving in apnea), and will withdraw the endotracheal tube right to the glottis. The surgeon punctures the trachea using an Abocath connected to a 10 cc syringe filled halfway with serum. The location in the airway will be checked by aspiration with the syringe that creates a bubble in the serum when entering the airway. The needle with the syringe is withdrawn and the Abocath is left within the lumen of the trachea (Fig. 1).

**Figure 2:**
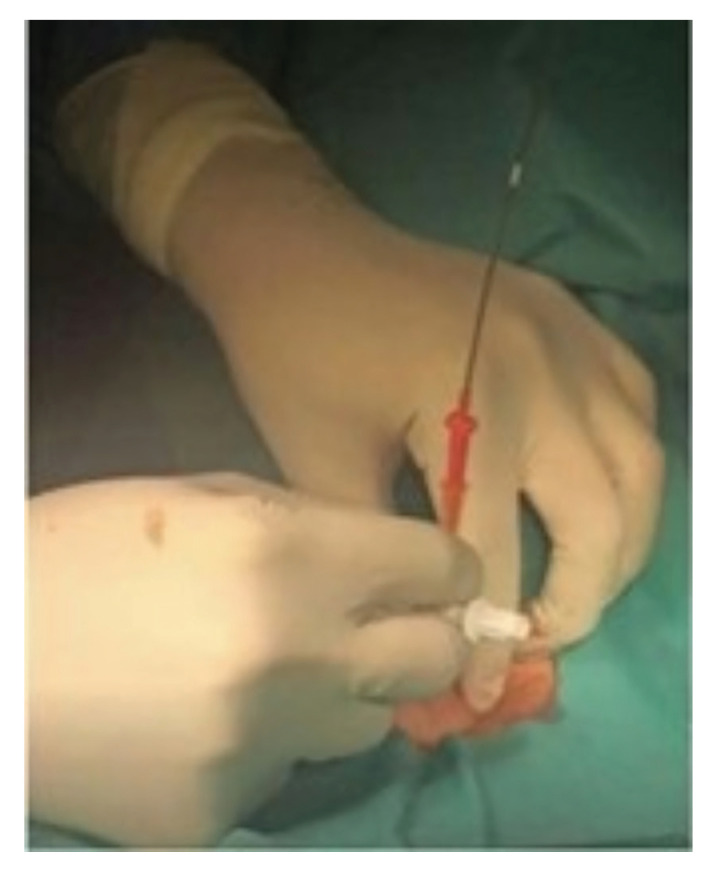
Insertion of endotracheal guide.

3- The guide is introduced through the Abocath, which, as its name indicates, will guide the way for the introduction of the dilators in a second step. Then the Abocath is removed (Fig. 2).

**Figure 3:**
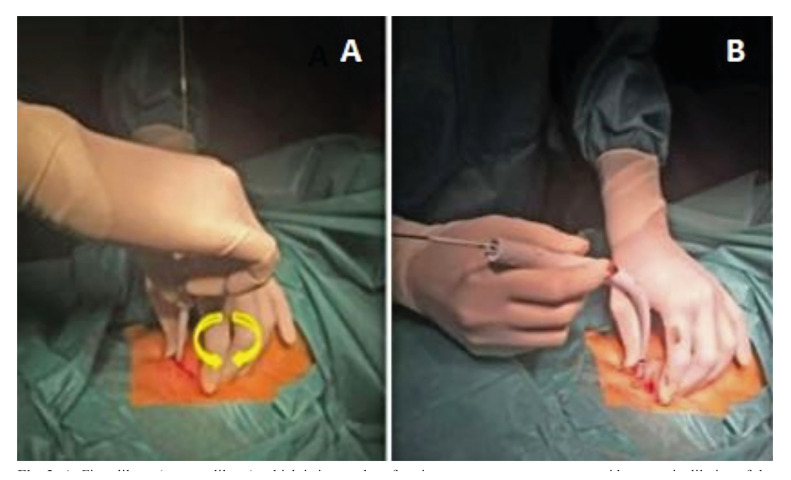
A. First dilator (narrow dilator), which is inserted performing rotatory movements to avoid traumatic dilation of the tracheal orifice; B. Second dilator (wide dilator).

4- Introduction of the first dilator, making rotational movements once introduced in the trachea (Fig. 3).

5- A wider and more rigid white guide catheter is placed, guided over the initial guide, and over everything the second dilator will be placed, which should be inserted until a black mark can be seen on the neck (Fig. 3).

**Figure 4:**
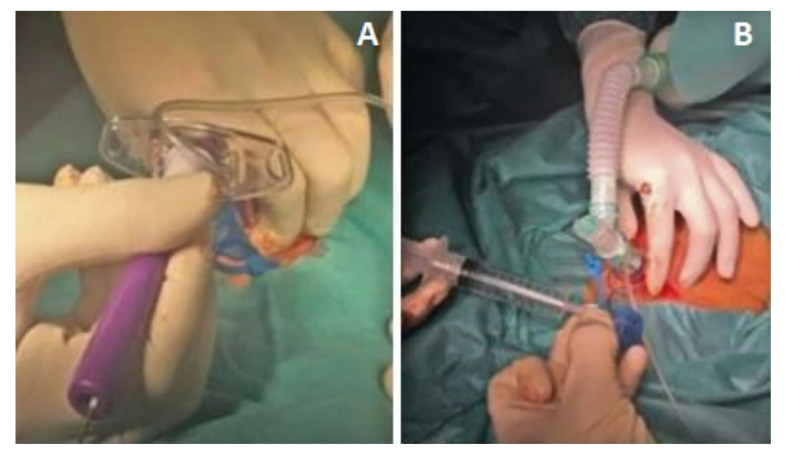
A. Introduction of tracheostomy cannula over the guide; B. Connection of cannula to mechanical ventilation. The cannula balloon is inflated to isolate the airway.

6- The tracheostomy cannula is advanced over the complex guide-catheter, and once it is placed inside the trachea, the guide, catheter and the final cannula´s guide are removed (Fig. 4).

7- The cannula´s shirt is inserted into the cannula and the pneumatic balloon is inflated. At this point, the patient is connected to mechanical ventilation. The intensivist / anesthetist, once adecuate ventilation has been verified, removes the rest of the endotracheal tube (Fig. 4).

At the end of the procedure, in our center we place a cellulose dressing around the tracheostome to perform local hemostasis.

## Results

According to the information panel of the John Hopkins Center, the infection mainly affects people between 30 and 79 years (87% of cases). Of those infected, 81% are asymptomatic or with mild symptoms, while 15% require hospitalization, and of these, 3-15% will need ventilatory support in an intensive care unit with mechanical ventilation or use of ECMO (extracorporeal membrane oxygenation) (7-9).

The main clinical aspects of the patients analyzed were:

1- Arterial hypertension -19 patients (54%).

2- Dyslipidemia - 17 patients (48.5%).

3- Mellitus diabetes - 11 patients. (31.5%).

4- Respiratory system disease (Asthma, COPD) - 9 patients (25.7%).

It should be noted that the presence of hypertension and dyslipidemia occurs in practically half of the patients in our study (Table 2)

According to the data collected in our study, the mean age of the patients who underwent a tracheostomy in the study period was 66 years old. 32 tracheotomies were performed in men and 3 in women (91.5% men versus 8.5% women). The mean time from orotracheal intubation to percutaneous tracheostomy was 24 days, and the mean time to decannulation from tracheostomy was 25.6 days. The American Academy of Otolaryngology recommends considering a tracheostomy in patients with sTable pulmonary status, preferably after the second or third week of intubation.

**Table 2:**
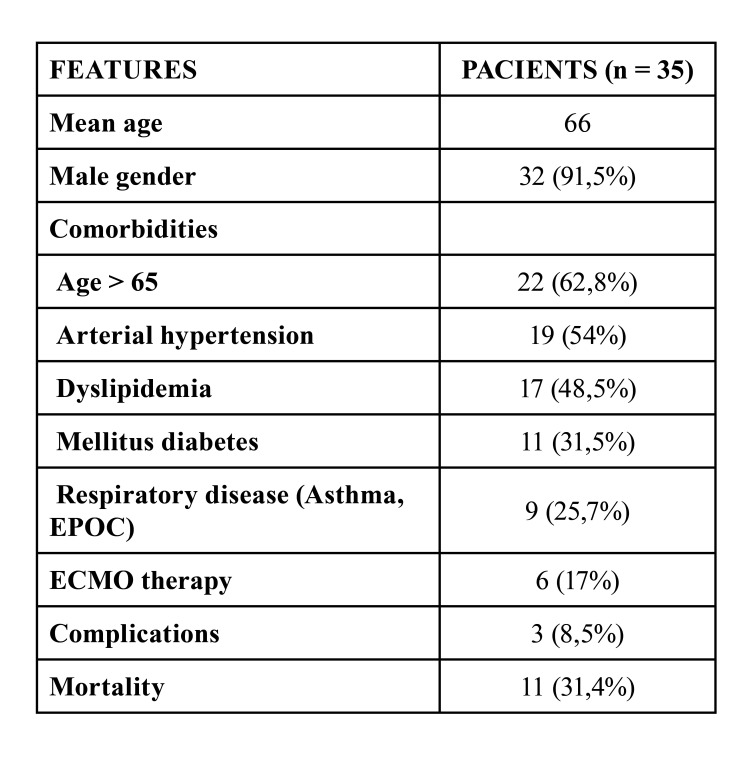
The data in the table show the distribution of patients in relation to the different features analyzed.

17% of the patients admitted to the intensive care unit were candidates for ECMO therapy (6 of 35 patients).

Of the 35 patients evaluated, 31.4% resulted in death (11 patients). Two of them were on ECMO therapy. Of the patients who were discharged, one died a month later due to respiratory complications.

The death occurred at 46.25 days on average from the performance of the tracheostomy, and the mean time to discharge from hospital was 52 days.

In similar studies such as the one carried out at the Hospital de La Paz (J. L. Del Castillo Pardo de Vera *et al*, 2020) on 22 patients infected with COVID-19 who underwent open tracheostomy, their results show similar Figures of death (27, 27%), but the mean time to perform the tracheostomy was minor (a mean of 11 days).

100% of the procedures were performed without electrocautery material, since diathermy could produce small particles that serve as a vehicle to spread the virus (10,11).

As complications, only 3 bleeds around the tracheostoma were recorded, not during the surgery but postoperatively, which were resolved with local hemostatic measures (use of absorbable cellulose hemostatic). No other complications such as infections, tracheal ruptures, fistulas or deaths were observed, complications only being observed in 8.5% of the cases. No emergency tracheostomy were performed.

Studies carried out in other centers show some similar data, as it is shown by the results observed in 50 COVID patients from the Otorhinolaryngology service of the Hospital Clínic de Barcelona who underwent tracheostomy (12), where a mean age of 63.8 years was observed, with a male / female ratio of 2 / 1. An apnea protocol was carried out at the time of entering the trachea, similar to the one of our center, in up to 80% of cases, without being necessary to perform any emergency tracheostomy either. The complications after the intervention were minimal, presenting bleeding in 6 patients (12% of the cases) controlled with local haemostasis measures. In a mean follow-up period of 73.8 days, 8 patients died due to complications from COVID (16%).

Similar are the data provided by the New York University Langone Health Manhattan's Campus (13), in a study of 98 percutaneous tracheostomies performed on COVID-19 patients, in which they perform a technique in order to reduce the risk of exposure during tracheostomy. It is based on the introduction of the fiberscope next to the endotracheal tube, and not inside it, stopping the ventilation when deflating the balloon of the endotracheal tube and reintroducing it once it has been inflated again. The average age was 57 years, with 82% males. Of the total number of patients, 7% died due to respiratory complications, and only 5% presented complications (all of them bleeding, resolved with local hemostasis in the first 48 hours). Staff was not infected.

Other studies compare the use of open versus percutaneous surgical tracheostomy, such as the one performed by the South London Adult Critical Care Network, which includes a total of 201 patients infected with COVID-19 who underwent tracheostomy (126 percutaneous, 77 surgical technic), comparing the ratio of perisurgical complications, mean decannulation time and mortality, not finding significant differences in both groups (14).

In our center, characteristically, we perform all the procedures keeping the patient under apnea from the opening of the airway to the placement of the cannula and reconnection to the ventilator, without causing any contagion in the staff in charge of performing the procedure.

## Discussion

Tracheostomy is a high-risk procedure, but it is a necessary technique in COVID patients who, due to their situation, require intubation to maintain an adequate ventilatory situation and sedation state.

SARS-CoV-2 is transmitted from person to person by respiratory route through Flügge drops (> 5 microns) and aerosols originated by the infected person (15), which represents an increased risk for procedures that involve opening of the airway, such as tracheostomy (16). That is why, despite being a procedure that improves the situation of the intubated patient, both reducing the complications associated with prolonged intubation, and allowing greater ease at the time of weaning, it is not a risk-free procedure.

To minimize it, we conclude that performing the tracheostomy using protective measures (use of PPE, performing a tracheotomy on the patient's bed without requiring displacement, minimizing the number of people involved in performing the procedure, and performing percutaneous tracheostomy in apnea situation, which allows rapid performance of the tracheostomy, maintaining apnea during tracheal dilation and insertion of the cannula), allows the patient to be kept in a more favorable ventilatory situation, with minimal risk of exposure to the healthcare staff who perform the procedure.

As guidelines show (17), the intraoperative exposure time to aerosolized secretions should be the minimum. This can be achieved in the following way: relaxing the patient previously to the intervention to minimize coughing, keeping the patient apnea before entering inside the airway and avoiding the use of suction equipment.

According to the evidence, the percutaneous technique offers advantages in elective tracheotomies over the reduction in the appearance of certain peri and postoperative complications (tracheal stenosis, malacia ...), perhaps due to the lower tissue damage at the time of its performance, the use of dilators, and the possibility of performing it on the patient's bed in the ICU (18).

After performing tracheostomy, correct removal of personal protective equipment is very important, as described in the guidelines. For this, it is recommended to receive prior training about equipping and removing the PPE to reduce the risk of contagion (19).

Considering the results observed in our center, we can conclude that the performance of the percutaneous tracheostomy maintaining apnea during the dilation of the airway and the insertion of the cannula, all this with the minimum necessary staff, a correct use of PPE and performing the procedure in the same critical box minimizes and even avoids the presence of contagions in the staff involved in the procedure, and it has been demonstrated as a safe technique for the patient.

However, there is no information available about the superiority of either of the two tracheostomy techniques (surgical technic versus percutaneous) from the point of view of safety in infection transmission, suggesting that the staff performing the procedure carry it out in the way and with the material they are familiarized with (20).
